# Deep-learning-based recognition of multi-singularity structured light

**DOI:** 10.1515/nanoph-2021-0489

**Published:** 2021-10-14

**Authors:** Hao Wang, Xilin Yang, Zeqi Liu, Jing Pan, Yuan Meng, Zijian Shi, Zhensong Wan, Hengkang Zhang, Yijie Shen, Xing Fu, Qiang Liu

**Affiliations:** Key Laboratory of Photonic Control Technology (Tsinghua University), Ministry of Education, Beijing 100084, China; Department of Precision Instrument, State Key Laboratory of Precision Measurement of Technology and Instruments, Tsinghua University, Beijing 100084, China; Electrical and Computer Engineering Department, University of California, Los Angeles, CA 90095, USA; Optoelectronics Research Centre, University of Southampton, Southampton SO17 1BJ, UK

**Keywords:** deep learning, optical secret key sharing, orbital angular momentum, structured light, vortex beams

## Abstract

Structured light with customized topological patterns inspires diverse classical and quantum investigations underpinned by accurate detection techniques. However, the current detection schemes are limited to vortex beams with a simple phase singularity. The precise recognition of general structured light with multiple singularities remains elusive. Here, we report deep learning (DL) framework that can unveil multi-singularity phase structures in an end-to-end manner, after feeding only two intensity patterns upon beam propagation. By outputting the phase directly, rich and intuitive information of twisted photons is unleashed. The DL toolbox can also acquire phases of Laguerre–Gaussian (LG) modes with a single singularity and other general phase objects likewise. Enabled by this DL platform, a phase-based optical secret sharing (OSS) protocol is proposed, which is based on a more general class of multi-singularity modes than conventional LG beams. The OSS protocol features strong security, wealthy state space, and convenient intensity-based measurements. This study opens new avenues for large-capacity communications, laser mode analysis, microscopy, Bose–Einstein condensates characterization, etc.

## 1 Introduction

In addition to linear momentum, photons can carry angular momentum divided into two forms, spin and orbital [1, 2]. The spin angular momentum usually refers to light’s polarization [3], and the orbital angular momentum (OAM) arises if the wavefront is helically shaped with a phase singularity on the axis [4]. The typical unit of the helical phase is an azimuthal-dependent term ${\text{e}}^{i\ell \varphi }$ , where ℓ reveals topological charge (TC) and φ is the azimuthal angle [5]. In contrast to conventional vortex beams with single singularity, e.g. Laguerre–Gaussian (LG) and Bessel modes, recent advances highlight the general structured light with distributions of multiple singularities, such as singularity array [6, 7], vortex lattice [8], and SU(2) vortex geometric mode [9], [10], [11]. Vortex beams have triggered intense research over the past three decades [12] for their demonstrated potentials in both classical (optical tweezers [13, 14], holographic encryption [15], communications [16], measurements [17, 18], and nonlinear effects [19, 20]) and quantum applications (quantum secret sharing [21], quantum switches [22], quantum information processing [23], and quantum states tomography [24]). For most of these pertinent application scenarios using optical vortices, accurate detection of OAM is a basic premise, which may resort to interference [25], diffraction [26], log-polar transformation [27], spiral transformation [28], multiplane light conversion [29], and deep learning algorithms [30], to name a few.

However, the current detection methods mainly work for single-singularity vortices. The precise measurement of more complex multi-singularity counterparts still remains a demanding task. This task requires detecting all vortices first and quantifying each vortex then. On the other hand, all the topological information of optical vortices is stored in phase whereupon a relevant issue arises: can we decode the phase directly? Indeed, phase recovery in the presence of singularity is a long-standing bugbear [31, 32]. Researchers have invoked transport of intensity equation (TIE) [33], Gerchberg–Saxton-based algorithms [34], wavefront modulation [35, 36], diffractive imaging [37], [38], [39], or interferometer [40, 41], to retrieve the phases of vortex beams. However, these profound endeavors still suffer from requiring multiple high-precision (∼μm) axial intensity measurements and rigorous boundary conditions, or nonintuitive iterative algorithms, or much *a priori* knowledge, or being vulnerable to experimental noises. Most importantly, many methods are feasible for simple vortices only and the recovered performances are unfulfilling.

In this work, we propose a deep learning framework, which is dubbed “VortexNet”, to overcome these hurdles. It’s mention-worthy that recent years have witnessed transformative advances of deep neural networks in analyzing vortex light, spanning from scalar [30, 42], [43], [44], [45], [46], [47], [48], [49] to vectorial ones [50]. Most previous achievements regard single-singularity OAM recognition as a classification or a regression task. The outputs of their networks are only simple numbers, which relate to the exact TCs. However, we here handle it as a generative problem whereby VortexNet yields complex phases containing dozens of singularities in an end-to-end manner, thus unleashing rich and intuitive physics information to comprehend twisted photons. Here, we demonstrate the phase reconstruction approach onto a set of multi-singularity SU(2) vortex modes, which accommodate conventional LG vortex modes as simple members and characterize high-dimensional topological properties [51]. Benefitting from the satisfactory performance of VortexNet, a phase-based optical secret sharing (OSS) protocol is demonstrated as an exemplary application. VortexNet can acquire phases of SU(2) beams with mode accuracy up to 93.6% from only two measurements of intensity. It also works for LG modes even with large TCs and degenerate intensities as well as other general phase objects. Therefore, it is a universal and practical tool for many classical and quantum information processing systems.

## 2 Materials and methods

**Figure 1:**
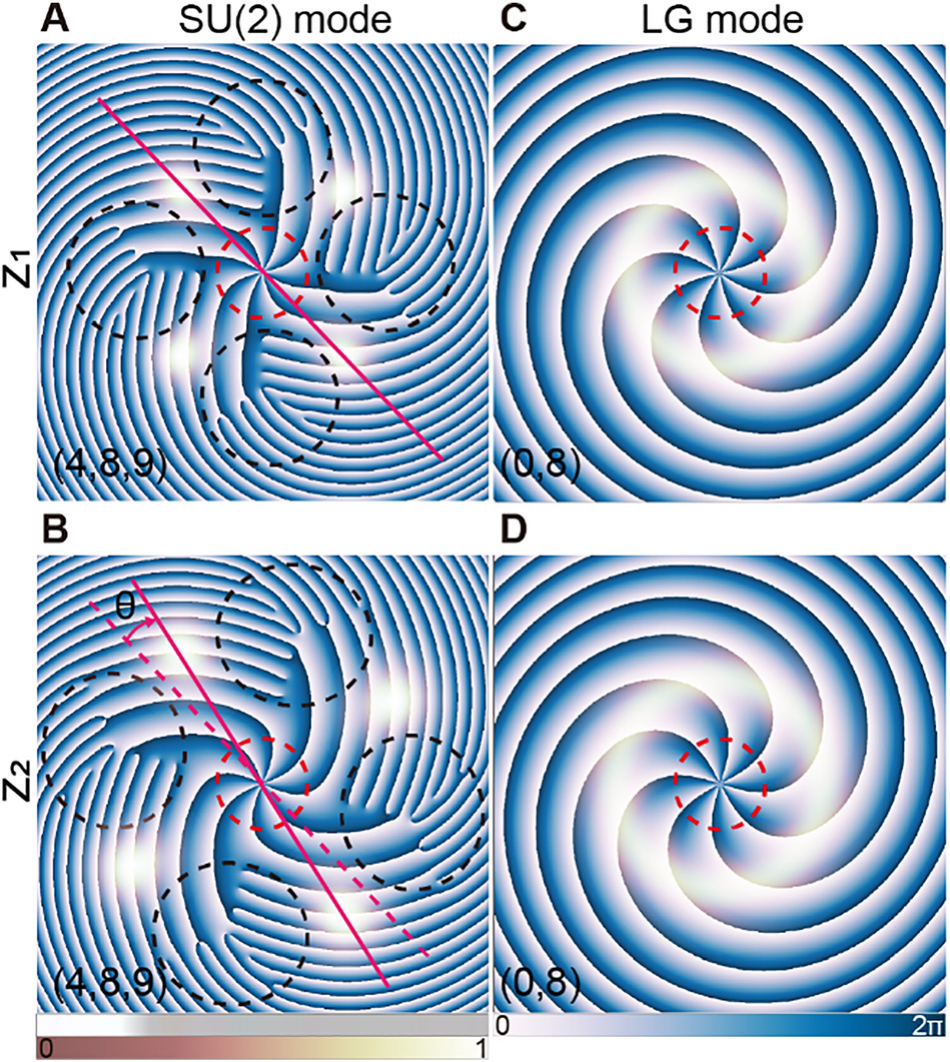
Comparison of SU(2) vortex mode (A)–(B) and LG mode (C)–(D) at two axial positions. Other than the single singularity of LG mode induced by central OAM as indicated by red circles, SU(2) mode characterizes more singularities shown by black circles. Besides, as SU(2) mode propagates from *z*_1_ (A) to *z*_2_ (B), its intensity undergoes an azimuthal shift *θ* while that of LG mode remains circularly symmetric. Note the intensity layers are set with opacity.

As a typical example of complex vortex beams with multiple singularities, SU(2) mode can be excited in a special resonator [52] and it can be launched by coherently superposing a set of LG modes,

(1) $$\begin{array}{l} \psi_{Q,n_{0},M}\left(x,y,z;\phi \right)\\ =\frac{1}{2^{M/2}}\overset{M}{\underset{K=0}{\sum }}{\left(\begin{array}{l} M\\ K \end{array}\right)}^{1/2}\exp\left(iK\phi \right)\psi_{0,n_{0}+Q\cdot K,s_{0}-P\cdot K}^{LG}\left(x,y,z\right)\text{,} \end{array}$$

where $\psi_{p,\ell ,s}^{LG}$ represents LG mode with radial index *p*, angular index *ℓ*, and longitudinal order *s*. Integer *Q* refers to how many folds in the rotational symmetry, *n*_0_ decides the TC of the axial vortex, *M* reveals its localized OAM of off-axis singularities and ϕ∈(0, 2π) determines the initial phase. Each SU(2) vortex mode corresponds to a pair of parameters (*Q*, *n*_0_, *M*) unraveling the topological information. By utilizing the multiple parameters of SU(2) modes, researchers successfully realized the classical analogy of high-dimensional quantum Greenberger–Horne–Zeilinger states recently [51]. Figure 1 illustrates the difference between circular-shaped LG mode (*p*, *ℓ*) = (0, 8) whose intensity always remains in circular symmetry upon propagation, and SU(2) mode (*Q*, *n*_0_, *M*) = (4, 8, 9) which is favored with large OAM, multiple singularities, helical star-shaped pattern and spiral propagation trajectory (see Supplementary material I for more details of SU(2) modes).

**Figure 2:**
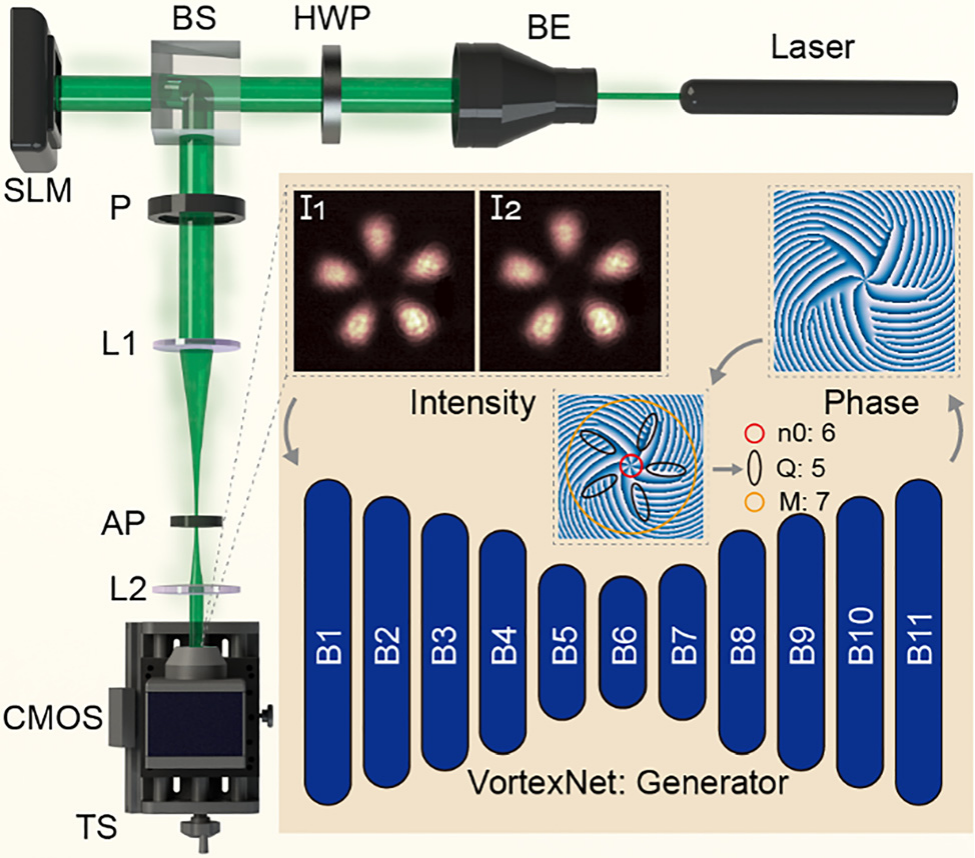
Schematic of experimental layout for the SU(2) mode creation and vortex phase acquisition. BE, beam expander; HWP, half-wave plate, which aligns the source to be p-polarized; BS, beam splitter, which assures the optical path structure to be rectangular; SLM, spatial light modulator; P, polarizer, which adjusts the light intensity; L1, L2, lens; AP, aperture, which filters out the first diffraction order; CMOS, camera; TS, translation stage, which moves the CMOS in the *z*-direction. After recording two intensity patterns with a longitudinal interval of 10.00 mm, the generator of VortexNet reconstructs the phase and then the value of the parameters (*Q*, *n*_0_, *M*) can be decoded accordingly. B, block, configurable unit of the generator part of VortexNet.

Inspired by TIE [32, 53], a prevailing benchmark model in phase retrieval assignments, wherein the phase at the target plane can be unwrapped given the target-plane intensity and intensity differential, we construct VortexNet to calculate phases based on *two* intensity patterns *I*_1_ and *I*_2_ as elucidated in Figure 2: one is that of target plane,

(2) $$\begin{array}{l} I_{1}={\left|\psi_{Q,n_{0},M}\left(x,y,z_{r};\phi \right)\right|}^{2}\text{,} \end{array}$$

where *z*_*r*_ is the Rayleigh distance. The other is the intensity pattern of the defocused plane,

(3) $$\begin{array}{l} I_{2}={\left|\psi_{Q,n_{0},M}\left(x,y,z_{r}+\Delta z;\phi \right)\right|}^{2}\text{,} \end{array}$$

where Δz is the diffraction distance after the target plane. In the experiment, the defocused light can be characterized by

(4) $$\begin{array}{l} \psi_{Q,n_{0},M}\left(x,y,z_{r}+\Delta z;\phi \right)=\frac{\exp\left(ik\Delta z\right)}{i\lambda \Delta z}\exp\left[i\frac{k\left(x^{2}+y^{2}\right)}{2\Delta z}\right]\\ \begin{array}{l} F{\left\{\psi_{Q,n_{0},M}\left(x_{0},y_{0},z_{r};\phi \right)\exp\left[i\frac{k\left(x_{0}^{2}+y_{0}^{2}\right)}{2\Delta z}\right]\right\}}_{f_{x}=\frac{x}{\lambda \Delta z},f_{y}=\frac{y}{\lambda \Delta z}}\text{,} \end{array} \end{array}$$

where *k* = 2*π*/*λ* is the wavenumber, *F*(∗) denotes the spatial Fourier transformation. As a notable example, we plot these two experimental patterns of mode (*Q*, *n*_0_, *M*) = (5, 6, 7) in the inset of Figure 2. With visible *I*_1_ and *I*_2_ as inputs at hand, VortexNet is expected to reconstruct the invisible phase of the target plane,

(5) $$\begin{array}{l} P_{Q,n_{0},M}\left(x,y,z_{r};\phi \right)=\arg\left[\psi_{Q,n_{0},M}\left(x,y,z_{r};\phi \right)\right]\text{,} \end{array}$$

where arg(∗) returns the argument of a complex variable.

To train the VortexNet, massive data samples are necessary. Rather than utilizing a laser cavity, which is challenging for efficient on-demand modes generation, we collect datasets via spatial light modulator (SLM) as illustrated in Figure 2. More specifically, a 532 nm laser (CNI Laser, MGL-III-532) is employed as the source of the Gaussian beam. After being expanded eightfold, it undergoes modulation of the SLM (Hamamatsu, X13138-04, resolution of 1280 × 1024, pixel size of 12.5 μm). The SU(2) state $\psi_{Q,n_{0},M}\left(x,y,z_{r};\phi \right)$ is encoded into the computer-generated-holographic mask through a complex amplitude modulation technique [54, 55]. Two lenses L1 (150 mm) and L2 (75 mm) constitute an optical 4-*f* system, through which the conjugate image of target vortex mode is recorded by the Complementary Metal-Oxide-Semiconductor (CMOS) camera (AVT Mako G-131B, resolution of 1280 × 1024, pixel size of 5.3 μm), which completes our first measurement. Then the CMOS is moved backward for 10.00 mm through a one-dimensional translation stage (GCM-830303M, resolution of 0.01 mm), leading to a weak diffraction of the SU(2) mode, the intensity of which is recorded as the second measurement.

VortexNet is inherently an adapted conditional generative adversarial network (GAN) (see Supplementary material III for network details), containing a generator and a discriminator [56, 57]. GANs are an instrumental category of deep neural networks with ever-improving results that can extract the hierarchical features of input data and enhance the prediction accuracy [58]. In this case, the generator outputs a “fake phase” and this output along with labeled phase (ground truth) are both fed into the discriminator. The discriminator strives to evaluate whether the input is real or fake. The two networks are trained jointly and they reach the “Nash equilibrium” when the training ends [59]. At this moment, the discriminator struggles to distinguish true or false and the generator returns a convincing phase.

## 3 Results

**Figure 3:**
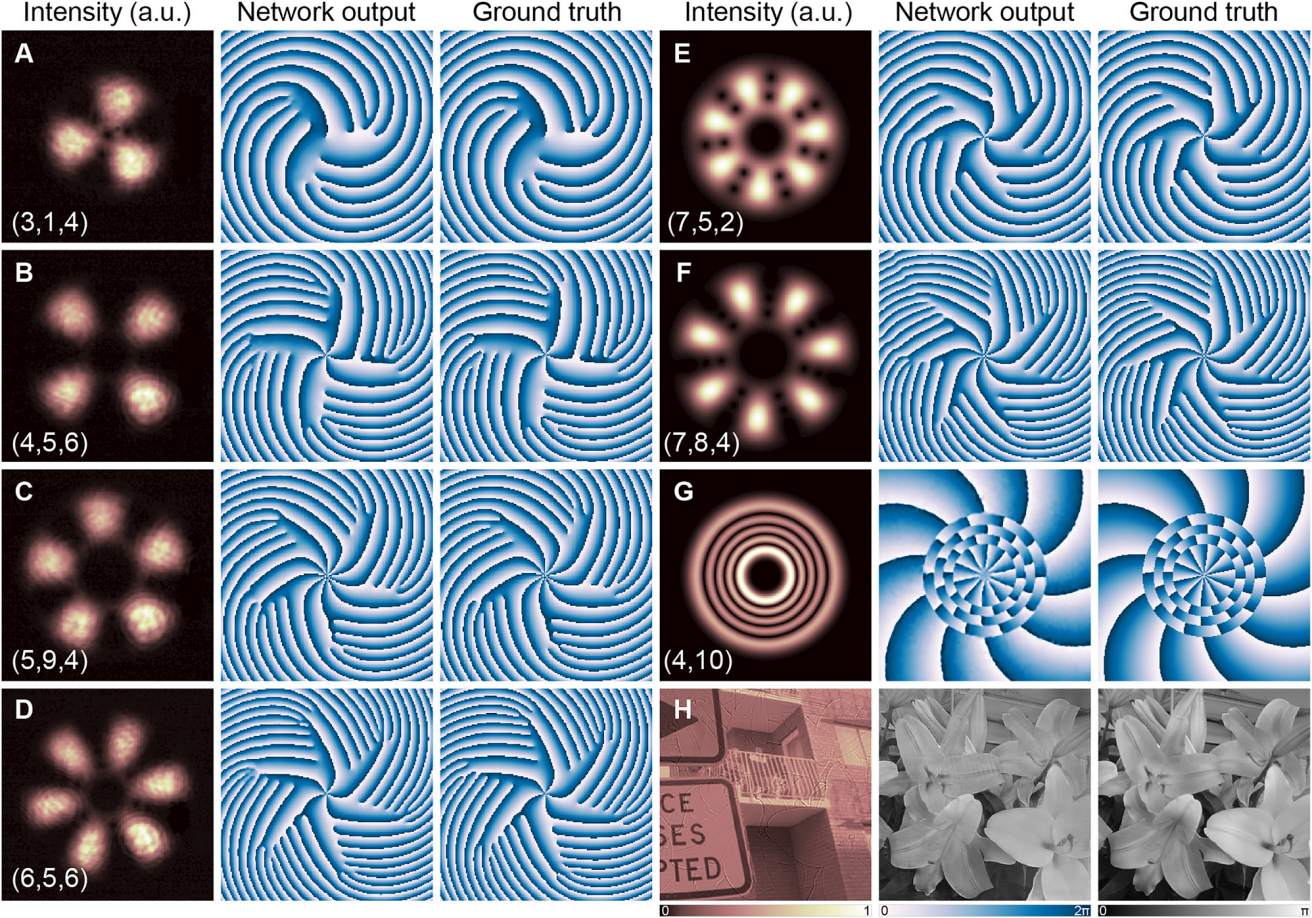
Characteristic inference results of VortexNet on the experimental test set (A)–(D), modes out of training, validation and test set (E)–(F), LG modes (G), and general phase objects (H). First and fourth columns: normalized intensity; Second and fifth columns: VortexNet output; Third and sixth columns: phase ground truth. More results are available in Supplementary material II.

**Figure 4:**
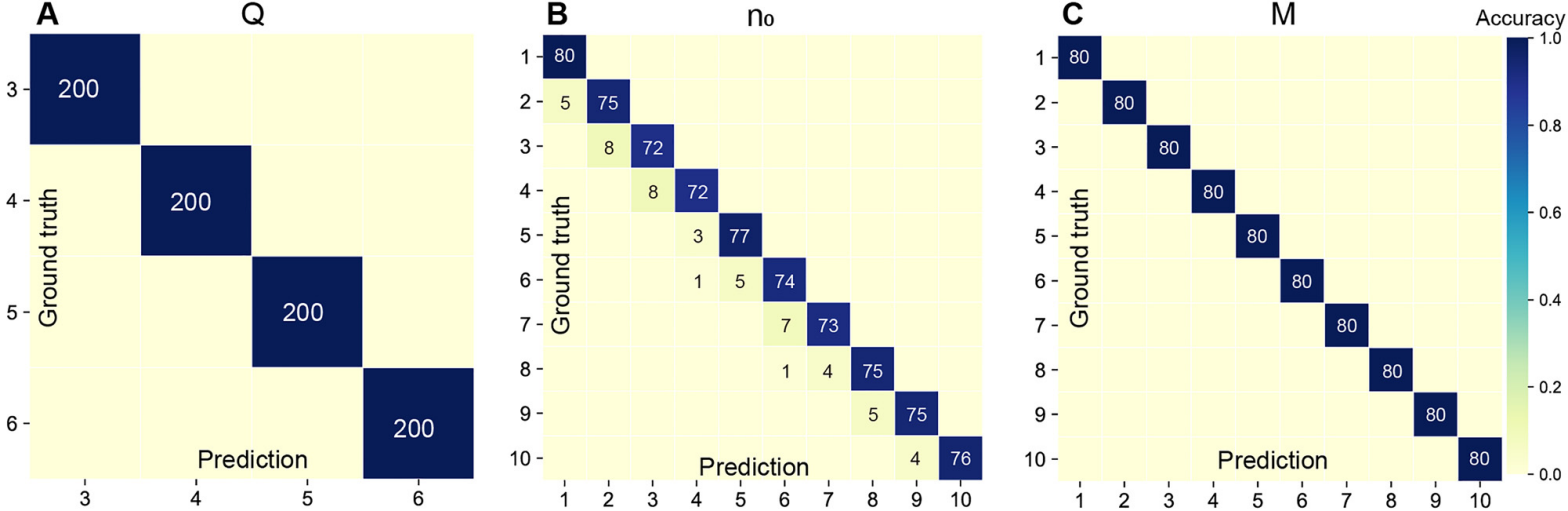
The confusion matrixes of experimental testing results, indicating the number of correctly (diagonal) and incorrectly (off-diagonal) predicted mode parameters (A) *Q*, (B) *n*_0_, and (C) *M*. Note the color of every box reflects the normalized accuracy.

The network is first trained using an experimental dataset with mode parameters *Q* ∈ {3, 4, 5, 6}, *n*_0_ ∈ {1,  2,  …, 10}, and *M* ∈ {1,  2, …,  10} (see Supplementary material II.A for dataset acquisition details). For each set of parameters (*Q*, *n*_0_, *M*), the initial phase *ϕ* ranges from 0 to 1.96*π* with an incremental value of 0.04*π*. There are overall 400 different states and 20,000 pairs of intensity images within the experimental dataset. Among them, we select 86% for training, 10% for validating, and 4% for testing the VortexNet. Some representative blind test results are shown in Figure 3(A)–(D), showing excellent agreement between the ground truth and network output (see more in Supplementary material II.B). Once the generator performs inference of the phase, one can decode the carried information of SU(2) mode. Based on three-phase read-out rules defined in Supplementary material I, the recognition accuracies of parameters *Q*, *n*_0_, and *M* on the test set are summarized in the confusion matrixes of Figure 4. The *accuracy* refers to the proportion of correctly classified modes and is calculated by comparing the reconstructed phase with respect to its corresponding ground truth. Accuracies of both *Q* and *M* reach 100% while that of *n*_0_ achieves 93.6%. Hence the overall mode accuracy of 93.6% is already obtained. To further evaluate the quality of generated phase structures quantitively, we calculate the peak-signal-to-noise (PSNR), structural similarity index (SSIM), and image correlation coefficient (CC) metrics. The average values of them are 14.89 dB, 0.75, and 0.76, respectively (see Supplementary material II.B for details). These values are not that prominent because of nonideal experimental conditions like power fluctuation of the laser source, lens glare, and CMOS noise. These imperfections are incorporated into the training process to beset the metrics of the reconstructed phase. However, benefitting from the read-out rules, some local flaws may degrade three metrics, they do not mislead us to identify the mode parameters and the mode accuracy of 93.6% is still impressive. We note that with an improved experimental system, the results can be enhanced. To validate this claim, we retrain a VortexNet based on exclusively simulated intensities and the mean PSNR, SSIM, and CC are improved to be 16.46 dB, 0.84, and 0.85, respectively. Remarkably, the recognition accuracy of (*Q*, *n*_0_, *M*) reaches 100%.

Later, to confirm VortexNet is learning to transform extracted features into phase structures rather than memorizing all the modes and overfitting the training set, we establish the second test set containing 64 states where $Q=7,\;n_{0}\in \left\{1,2,\ldots ,8\right\},\;M\in \left\{1,2,\ldots ,8\right\}$ , which separate from training, validation, and the first test set. We let the afore-trained model based on a digitally generated dataset to infer. Two characteristic results are depicted in Figure 3(E)–(F) with the overall accuracy reaching 93.75% and the average PSNR, SSIM, and CC being 12.00 dB, 0.57, and 0.58, respectively. This further supports the generalization of VortexNet’s capability to measure phase structures of various SU(2) modes once it is trained.

In order to analyze the physics-informed training phase of VortexNet, i.e. why two intensity measurements are inevitable, we naturally train a model with only *I*_1_ as input. It’s a strongly ill-posed inverse problem to retrieve $P_{Q,n_{0},M}\left(x,y,z_{r};\phi \right)$ and indeed the poor blind testing results in Supplementary material II.C bear this out. Interestingly, we train another VortexNet akin to TIE. More specifically, we now input *I*_1_ and a differential approximated as $I_{\text{diff}}=\left(I_{1}-I_{2}\right)/\Delta z$ . The convergence time and mode accuracy are similar to the model with *I*_1_ and *I*_2_ as input. It’s quite reasonable because a linear operation does not minish the amount of information and is easily learnable for deep neural networks.

In addition, we realize that this computational network not only can be applied for multi-singularity vortex modes but also more universal phase reconstruction missions. Part of the LG modes results as well as other general phase images based on simulated datasets are illustrated in Figure 3(G)–(H) (see more in Supplementary material II.D). It’s notable that VortexNet trained with the proper dataset can effectively solve the intensity degenerate problem that previous endeavors encountered [30, 43], where the intensities of $\psi_{p,\ell }^{LG}$ and $\psi_{p,-\ell }^{LG}$ are identical to make the previous network unable to distinguish.

**Figure 5:**
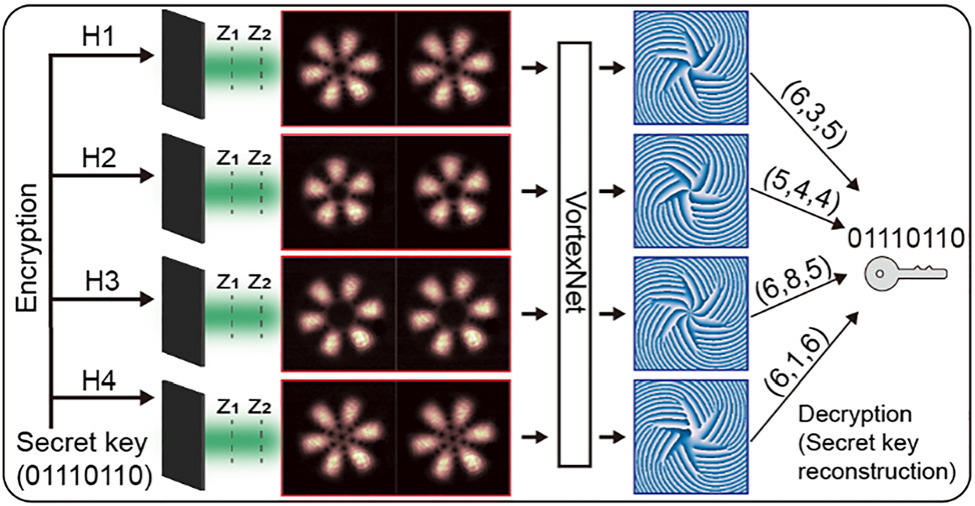
The phase-based OSS workflow. The secret key is encrypted into holograms (H1, H2, H3, and H4) that modulate the incoming light into distinct SU(2) modes. Each shareholder catches two intensities of SU(2) mode at positions *z*_1_ and *z*_2_. The decryption must involve honest collaboration and VortexNet to calculate the phases.

As we discussed before, VortexNet can directly deliver phases of SU(2) modes through indiscernible intensities hence it opens new pathways for utilizing high-dimensional topological properties in information dissemination. As data security becomes increasingly crucial, optical-based secret sharing thrives due to its abundant degrees of freedom, broad bandwidth, and susceptibility to eavesdropping [60, 61]. We, therefore, devise a phase-based OSS protocol enabled by VortexNet as depicted in Figure 5.

First, we allocate every mode (*Q*, *n*_0_, *M*) to an eight-bit binary number (see Supplementary material IV for detailed rules). Suppose the secret *S* is encoded as an eight-bit binary number (01110110 in this case) and in a traditional secret sharing scheme, the distributor will give three of four shareholders each a random eight-bit number (*N*_1_, *N*_2_, and *N*_3_). The last player has to get the result of $S⊕N_{1}⊕N_{2}⊕N_{3}$ , where ⊕ denotes bitwise exclusive or (XOR). Instead, to complicate the message, we allot a bundle of SU(2) lights to every player. Based on the experimental scheme in Figure 2, we implement the allocation operation through four holograms and in fact, this can also be implemented technically via one hologram that customizes four diffraction orders further [62]. Every player takes two shots of the incoming SU(2) beam as his/her share of the secret. To decrypt the secret key, firstly, all shareholders need to interpret two pieces of intensities through VortexNet, which is held in the hands of “the president”. Then “the president” honestly takes out their shares based on retrieved phases and adopts bitwise XOR operation. The high security stems from exotic and noninterpretable SU(2) intensity patterns and additional layers endowed by VortexNet. There are wealthy SU(2) states that are available to complicate the secret and enhance the security further. The intensity measurement is also easily realized in practical scenarios (see more analysis in Supplementary material IV).

## 4 Discussions and conclusions

The general significance of VortexNet covers several aspects: it is envisaged to recover phases of other multi-singularity beams such as vortex lattice [8], fractional vortex beam [63] as well as diverse categories of structured light—LG beam, Airy beam, and Bessel beam, to name a few—it liberates us by encoding/decoding *phase* information from only caring about intensity utilizations of shaped beams. The network structure can also be cascaded with another GAN which accommodates VortexNet’s phase as input and gives mode parameters directly [64]. This way the detection is reduced to be like Refs. [30, 50]. In experiments, the intensity interval is fixed to be 10.00 mm, which does not pose rigorous conditions for implementation. And the network is robust against small misalignment. In this work, we implemented our approach in steady laboratory conditions. In future research, it is meaningful to explore whether the adverse effects of atmospheric turbulence, ambient occlusions, or diffusers on structured light can be mitigated by an improved VortexNet without recourse to a probe beam [65]. In addition, VortexNet can also be enforced on complex amplitude reconstruction assignments, which can aid in digital holographic imaging [66]. Besides, the OSS is scalable both in terms of SU(2) modes number and secret-splitting rule. The first point concerns that as neural networks nowadays progress toward bigger model architecture, bigger dataset, and stronger computation power, one can scale up the SU(2) modes and construct a benchmark dataset/library for more sophisticated encryption. Secondly, the proposed OSS can be readily extended to (*t*, *n*)-threshold scheme where *t* or more of *n* players can decipher the key, and also grafted onto classical Shamir’s scheme, the Chinese remainder theorem-based scheme, etc. Furthermore, one can adopt the affluent SU(2) states to transmit information in a mode-multiplexing or shift-keying manner [67, 68]. Notably for structured light laser specialists, when a frequency-degenerate resonator emits SU(2) modes in the lab, one has to blindly select (*Q*, *n*_0_, *M*) in simulation to find the most similar one to characterize the emission mode due to noninterpretable intensity pattern before. Now VortexNet raises a hand to do the mode analysis job, which greatly facilitates the development of structured beam lasers [69, 70]. Last but not the least, the VortexNet-based techniques may also find further applications on electronic, X-ray as well as acoustics systems.

To conclude, we demonstrate a new approach to tackling structured beams enabled by deep learning, in particular phases with single/multiple singularities. The topological properties are revealed directly, accurately, and robustly by using only two convenient intensity-based measurements as inputs, even in the presence of experimental noises and instabilities. Empowered by SU(2) vortex modes with high-dimensional topological properties and great state space, numerous schemes employing them as information carriers are feasible now and we demonstrate a novel OSS protocol as an instance. This DL-assisted platform may promise relevant implications in large-capacity communications [48], laser mode analysis [69], microscopy [71], Bose–Einstein condensates characterization [72, 73], etc.

## Supplementary Material

Supplementary Material Details
