# Supplementary material for: Deep-learning-based recognition of multi-singularity structured light
Source: Nanophotonics. 2021 Oct 14;11(4):779–86. doi: 10.1515/nanoph-2021-0489 (PMC11501744; doi:10.1515/nanoph-2021-0489)
Supplement: Supplementary file 1 — Supplementary Material Details [file j_nanoph-2021-0489_suppl.docx]

Supplementary Materials

Hao Wang^†^, Xilin Yang^†^, Zeqi Liu, Jing Pan, Yuan Meng, Zijian Shi, Zhensong Wan, Hengkang Zhang, Yijie Shen*, Xing Fu* and Qiang Liu*

Deep-learning-based recognition of multi-singularity structured light

**Author information:**

^†^H.W. and ^†^X.Y. contributed equally to this work.

**^*^Corresponding authors: Yijie Shen:** Optoelectronics Research Centre, University of Southampton, Southampton SO17 1BJ, UK, E-mail: Y.Shen@soton.ac.uk

**Xing Fu and Qiang Liu**, Key Laboratory of Photonic Control Technology (Tsinghua University), Ministry of Education, Beijing 100084, China; State Key Laboratory of Precision Measurement of Technology and Instruments, Department of Precision Instrument, Tsinghua University, Beijing 100084, China, E-mail: [fuxing@tsinghua.edu.cn](mailto:fuxing@tsinghua.edu.cn); [qiangliu@tsinghua.edu.cn](mailto:qiangliu@tsinghua.edu.cn)

**Hao Wang, Zeqi Liu, Jing Pan, Yuan Meng, Zijian Shi, Zhensong Wan, Hengkang Zhang:** Key Laboratory of Photonic Control Technology (Tsinghua University), Ministry of Education, Beijing 100084, China; State Key Laboratory of Precision Measurement of Technology and Instruments, Department of Precision Instrument, Tsinghua University, Beijing 100084, China, E-mail: h-wang20@mails.tsinghua.edu.cn

**Xilin Yang:** Electrical and Computer Engineering Department, University of California, Los Angeles, California 90095, United States, E-mail: [mikeyangxl@outlook.com](mailto:mikeyangxl@outlook.com)

# Background of SU(2) Modes

We here outline the detailed physical background of SU(2) beams. In most cases of laser technology, the well-known Hermite-Gaussian (HG) modes under the Cartesian coordinate system (or Laguerre-Gaussian (LG) modes under Cylindrical coordinate system) are expected to oscillate as eigenmodes $\psi_{n,m,s}$ (or $\psi_{p,l,s}$) by elaborately controlling pumping position [1] and astigmatism [2], satisfying the Helmholtz equation: ${(\nabla}^{2}+k_{n,m,s}^{2})\psi_{n,m,s}\left( x,y,z \right)=0$, where $k_{n,m,s}$ is the eigenvalues, $(n,m)$ and $s$ are the orders of transverse and longitudinal modes respectively [3]. Under the context of paraxial approximation, the solution in Cartesian coordinate can be acquired by variable separation method as:

$$\begin{aligned} \psi_{n,m,s}^{\mathrm{HG}}\left( x,y,z \right)=\frac{C_{n,m}^{HG}}{w\left( z \right)}\exp\left[ -\frac{x^{2}+y^{2}}{w^{2}\left( z \right)} \right]H_{n}\left( \frac{\sqrt{2}x}{w\left( z \right)} \right)H_{m}\left( \frac{\sqrt{2}y}{w\left( z \right)} \right)\exp\left[ ik_{n,m,s}z+\frac{ik_{n,m,s}r^{2}}{2R\left( z \right)}-i\left( m+n+1 \right)\vartheta\left( z \right) \right],\#\left( S1 \right) \end{aligned}$$

where $C_{n,m}^{HG}=1/{\sqrt{\pi2^{n+m-1}n!m!}}$ is the normalization coefficient, $w\left( z \right)=w_{0}\sqrt{1+\left( z/{z_{R}} \right)^{2}}$ is beam radius with waist radius $w_{0}=\sqrt{\lambda{z_{R}}/\pi}$ and Rayleigh range $z_{R}$, $H_{n}(\cdot)$ is the Hermite polynomials of the $n$-th order, wavenumber $k_{n,m,s}=2\pi{f_{n,m,s}}/c$, $f_{n,m,s}$ is the eigenmode frequency, $c$ is the light speed, $\lambda$ is the wavelength, $R\left( z \right)=z+{z_{R}}/z$ and $\vartheta\left( z \right)={tan}^{-1}(z/{z_{R}})$ is the Gouy phase accumulated by propagation. The eigenmode frequency of the resonator can be expressed as $f_{n,m,s}=s\cdot\Delta f_{L}+(n+m+1)\cdot\Delta f_{T}$, where $\Delta f_{L}$ ($\Delta f_{T}$) is the longitudinal (transverse) mode spacing. However, when a laser cavity is operated in a frequency-degenerate state of ${\Delta f_{T}}/{\Delta f_{L}=P/Q}$, where $P$and $Q$ are two coprime integers, namely a series of (HG) eigenmodes with different orders but at the same eigenmode frequency coexists in a single optical resonator, the inherent mode bounces back and forth along a ray-like periodic orbit [4]. The trajectory’s shape and the number of bounces are dominated by the cavity length $L$, the cavity mirror curvatures $R$, and the position of the pump light. More specifically, the longitudinal mode spacing is given by $\Delta f_{L}=c/{2L}$ when the minor difference between the physical length and the geometric length can be neglected and the transverse mode spacing is given by $\Delta f_{T}={\Delta f_{L}\vartheta\left( L \right)}/\pi$. The mode-spacing ratio $\Omega=P/Q=(1/\pi)\cos^{-1}(\sqrt{1-L/R})$ in a plane-concave hemisphere cavity signifies the degeneracy, which is a rational number and varies in the range between $0$ and $1/2$ by changing the cavity length as $0<L<R$. As a result, the output mode is actually the coherent superposition of multiple degenerate eigenmodes, characterizing ray-wave duality [4,5]. The reason why it is referred as “SU(2) beam” or “SU(2) wavepacket” is that the frequency-degenerate effect leads the laser mode to perform like a quantum coherent state. As the derivation from Refs. [3,5-8], under the Schwinger (Bosonic) representation of the SU(2) algebra, the quantum coherent state is generally expressed by

$$\begin{aligned} |\left. \tau\right\rangle=\left( 1+\left| \tau\right|^{2} \right)^{-M/2}\sum_{K=0}^{M} {\binom{M}{K}}^{1/2}\tau^{K}|\left. K,M \right\rangle,\#\left( S2 \right) \end{aligned}$$

where $M$ is the total bosons number, the states $|\left. K,M \right\rangle$ are the states with $K$ bosons in the first mode and $(M-K)$ bosons in the second mode. For convenience, $\tau$ is rewritten as the normalized argument form $\tau=exp(i\phi)$ where $\phi$ is called the coherent state phase representing the SU(2) coherent state as

$$\begin{aligned} |\left. \phi\right\rangle=\frac{1}{2^{M/2}}\sum_{K=0}^{M} {\binom{M}{K}}^{1/2}\exp(iK\phi)|\left. K,M \right\rangle.\#\left( S3 \right) \end{aligned}$$

Notably, the basic state $|\left. K,M \right\rangle$ should be the eigenstate of the two-dimensional quantum harmonic oscillator with Hamiltonian of:

$$\begin{aligned} \hat{H}=\frac{{\hat{p_{x}}}^{2}}{2m_{x}}+\frac{{\hat{p_{y}}}^{2}}{2m_{y}}+\frac{m_{x}\omega_{x}^{2}x^{2}}{2}+\frac{m_{y}\omega_{y}^{2}y^{2}}{2}.\#\left( S4 \right) \end{aligned}$$

The HG modes perfectly satisfy the eigenstates constituting a complete orthogonal basis set in Hilbert space with the relationship of $\omega=\sqrt{{2\hbar}/{(m_{x}\omega_{x})}}=\sqrt{{2\hbar}/{(m_{y}\omega_{y})}}$ to fulfill the Schrödinger equation $\hat{H}|\left. \psi\right\rangle=E|\left. \psi\right\rangle$. So the coherent state under the SU(2) symmetry group is highly appropriate for the analogous description of the afore-discussed laser mode:

$$\begin{aligned} \psi_{Q,n_{0},M}\left( x,y,z;\phi\right)=\frac{1}{2^{M/2}}\sum_{K=0}^{M} {\binom{M}{K}}^{1/2}\exp\left( iK\phi\right)\psi_{n_{0}+Q\cdot K,0,S_{0}-P\cdot K}^{\mathrm{HG}}\left( x,y,z \right).\#\left( S5 \right) \end{aligned}$$

It is well known that an astigmatic mode converter can transform HG mode into LG mode, which possesses orbit angular momenta (OAM) [9]. Accordingly, planar SU(2) beam $\psi_{Q,n_{0},M}\left( x,y,z;\phi\right)$ will switch to circular SU(2) beam with a skewed trajectory carrying OAM after two cylindrical lenses i.e. SU(2) vortex mode, which reads

$$\begin{aligned} \psi_{Q,n_{0},M}\left( x,y,z;\phi\right)=\frac{1}{2^{M/2}}\sum_{K=0}^{M} {\binom{M}{K}}^{1/2}\exp\left( iK\phi\right)\psi_{0,n_{0}+Q\cdot K,S_{0}-P\cdot K}^{LG}\left( x,y,z \right),\#\left( S6 \right) \end{aligned}$$

where $\psi_{0,n_{0}+Q\cdot K,S_{0}-P\cdot K}^{\mathrm{LG}}(x,y,z)$ denotes LG mode given as

$$\begin{aligned} \psi_{p\mathcal{,l,}s}^{LG}=\frac{C_{p\mathcal{,l}}^{LG}}{w\left( z \right)}\left( \frac{\sqrt{2}r}{w\left( z \right)} \right)^{\left| \mathcal{l} \right|}\exp\left[ -\frac{r^{2}}{w^{2}\left( z \right)} \right]L_{p}^{\left| \mathcal{l} \right|}\left( \frac{2r^{2}}{w^{2}\left( z \right)} \right)\exp\left( i\mathcal{l}\theta\right)\exp\left[ ik_{n,m,s}z+\frac{ik_{n,m,s}r^{2}}{2R\left( z \right)}-i\left( 2p+\left| \mathcal{l} \right|+1 \right)\vartheta\left( z \right) \right],\#\left( S7 \right) \end{aligned}$$

where $C_{p\mathcal{,l}}^{LG}=\sqrt{{2p!}/\left[ \pi\left( p+\left| \mathcal{l} \right| \right)! \right]}$ is the normalization coefficient, $\mathcal{l}$ is the topological charge and other parameters follow the same definitions as that of HG mode. The right hand of Eq. S6 is the core structured light in this work. Usually, the value of $P$ is fixed to be 1 and $Q$ is set to be greater than 2.

To have a more vivid picture of SU(2) vortex mode’s characteristics, we give a comparison between it and LG mode in Figure S1. It can be seen that as they propagate, SU(2) mode goes along a spiral trajectory as opposed to that of LG mode. More importantly, the topological property of SU(2) mode is far more sophisticated or mysterious. So we introduce three rules to read out the corresponding parameter values from its phase. According to our empirical observation of these modes, the phase read-out rules are summarized as follows:

1. Count how many $2\pi$ jumps at the center (see the red circle of Figure S1 lower left) to determine $n_{0}$;
2. Count how many rib-like phase structures (See the black ellipse of Figure S1 lower left) in the image to retrieve $Q$;
3. Suppose that the count of $2\pi$ jumps around the peripheral circle (See the orange circle of Figure S1 lower left) is $N$ then the parameter $M$can be calculated as $M={(N-n_{0})}/Q$.

Note that considering the reconstructed phase image may exist local defects that are trivial to correctly recognize $(Q,n_{0},M)$, we here relax the third rule to be $M=Round[{(N-n_{0})}/Q]$. As the example in Figure S1 shows, one needs to first count the central OAM and obtain $n_{0}=10$. Then calculate how many rib-like structures are in the phase image as denoted by the black ellipse and obtain the rotational symmetry parameter $Q=5$. At last, one counts the overall $2\pi$ jumps along the outward orange circle as $N$ then the parameter $M$is decided by $M=\left( N-n_{0} \right)/Q$, i.e. $M=\left( 60-10 \right)/5=10$.


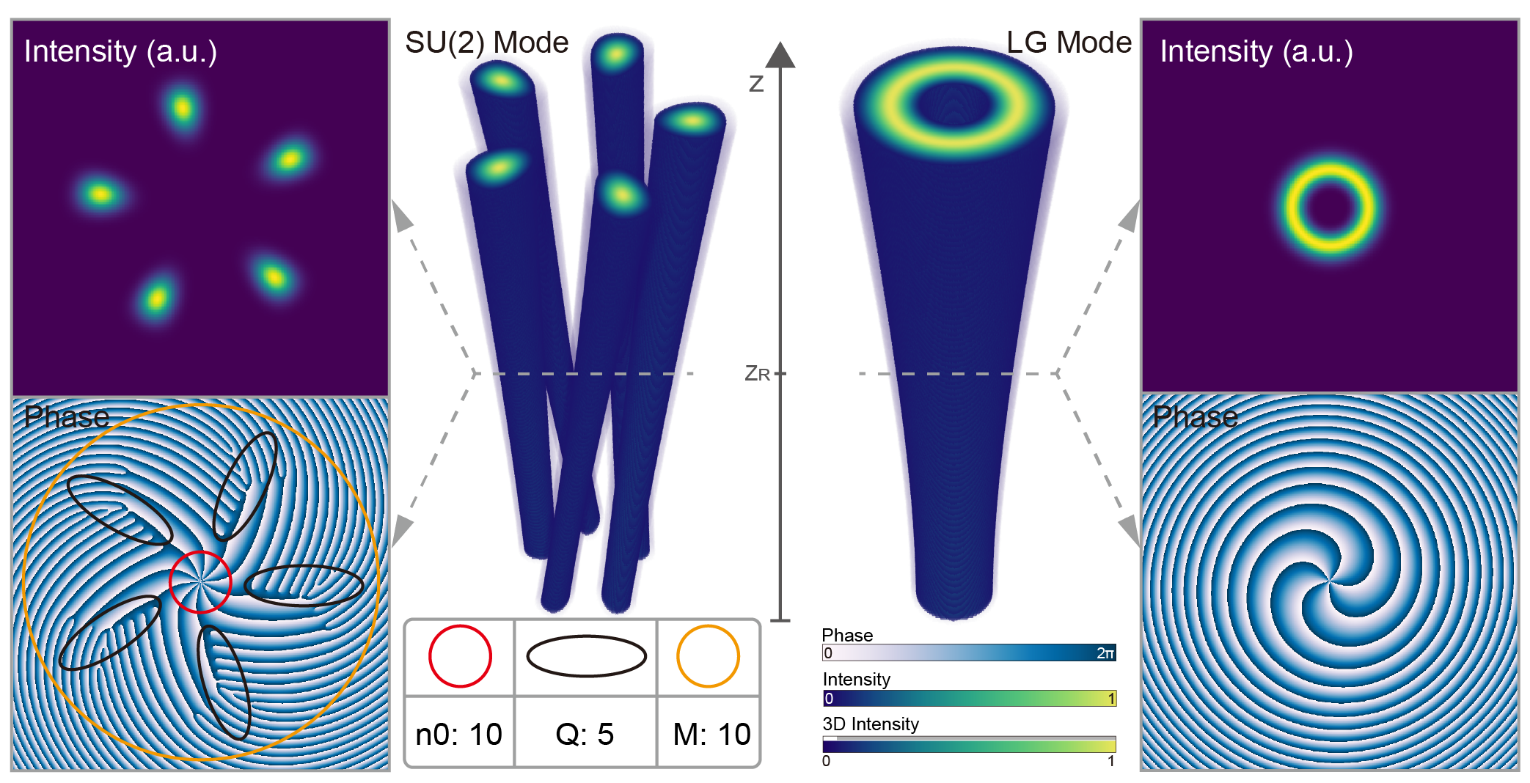


**Figure S1:** The comparison of SU(2) vortex mode (Left) and LG mode (Right). The lower left also illustrates how to decode the $(Q, n_{0},M)$ from the phase. Note the three-dimensional (3D) intensities are set with opacity.

# Dataset acquisition & results

## Data acquisition and preprocessing

In experiments, we design 20000 holograms containing 400 modes with different $(Q,n_{0},M)$ in total. Each mode $(Q,n_{0},M)$ corresponds to 50 initial phases where 43 of them are used for training, 5 of them are for validation and 2 of them are for testing. For each light field after uploading its matching hologram, we need to capture two intensities via a CMOS using a longitudinal translation stage, so there are 40000 intensity samples in the dataset. We implement the measurement in two steps with one CMOS rather than one step with two CMOSs, considering the increasing complexity of the experiment system. However, this may result in the magnification of laser source power instability issue because we will have to let it run for hours in the experiment. To alleviate this instability as well as noise effect, we measure 15 noise outputs in different times at each position. The noise output means that we upload nothing on the SLM then capture the intensity image. Once we obtain 40000 intensity samples and 30 noise outputs, we first calculate the average noise at each position and then we subtract the average noise from the samples in an element-wise manner. The resolution of our camera (AVT Mako G-131B) is 1280×1024, so after eliminating the noise, we select a square region of interest roughly (i.e. by naked eye rather than registration algorithm) and acquire a 1024×1024 pixel region. Then we downsample the data into 256×256 via bicubic interpolation method [10] to meet the VortexNet input requirement. It is noted that we normalize the data globally to maintain the holistic intensity variance of different modes.

## Demonstration of more experimental results

Despite the aforementioned data preprocessing, we find that our obtained experimental intensities are still not that “perfect”, as illustrated in the second and fourth columns of Figure S2. Compared to simulated ones, the overall size and uniformity of SU(2) intensity are available for further improvement with a better experimental environment and equipment. Surprisingly, the VortexNet can still work in this context.


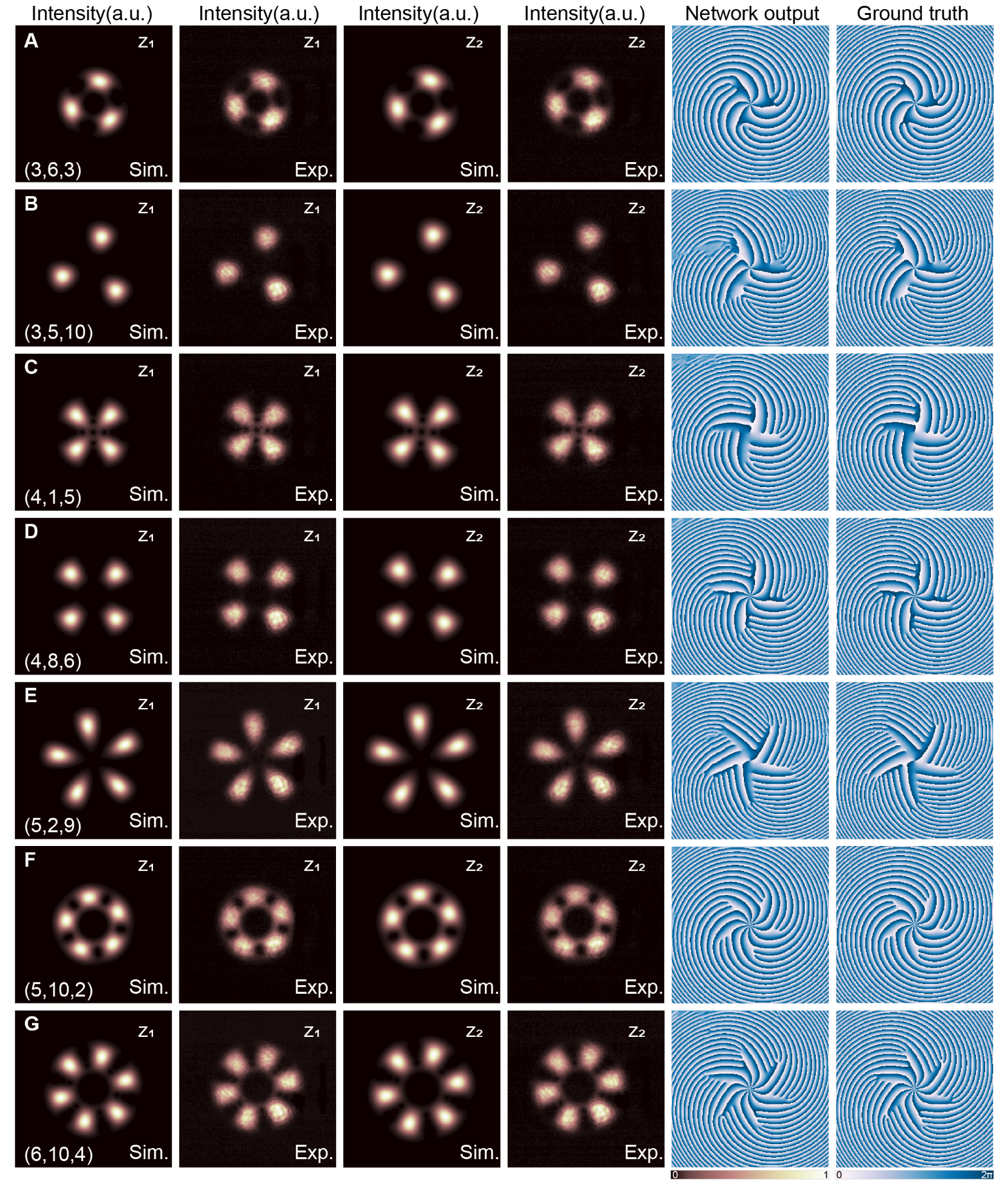


**Figure S2:** More simulated (First, third and sixth columns) and experimental (Second, fourth and fifth columns) inputs and outputs of VortexNet.

Another point worth mentioning is that we allow the existence of some local imperfections in the output phase image since they don’t mislead us to wrong parameter values as shown in the fifth column of Figure S2. These local flaws however, will degenerate the selected figure-of-merit in the main manuscript, peak-signal-to-noise (PSNR), structural similarity index (SSIM) and two-dimensional correlation coefficient (CC), more specifically. The PSNR is defined as

$$\begin{aligned} PSNR=10\log_{10} \left( \frac{{MAX}^{2}}{MSE} \right),\#\left( S9 \right) \end{aligned}$$

where $MAX$denotes the maximum value of calculated image and $MSE$ is the mean square error between the network output and the ground truth. The unit of PSNR is dB and the greater it is, the better the network output is. The SSIM is defined as

$$\begin{aligned} SSIM=\frac{\left( 2u_{t}u_{o}+C_{1} \right)\left( 2\sigma_{to}+C_{2} \right)}{\left( u_{t}^{2}+u_{o}^{2}+C_{1} \right)\left( \sigma_{t}^{2}+\sigma_{o}^{2}+C_{2} \right)},\#\left( S10 \right) \end{aligned}$$

where $u_{t}$ and $u_{o}$ represent the mean values of phase ground truth and network output, $\sigma_{t}^{2}$ and $\sigma_{o}^{2}$ represent the variance values of phase ground truth and network output, $\sigma_{to}$ denotes their covariance, and $C_{1}, C_{2}$ are two empirical hyperparameters. The SSIM value ranges from 0 to 1 and when the two images are the same, it equals to 1. The two-dimensional correlation coefficient is calculated by

$$\begin{aligned} CC=\frac{\sum_{i=1}^{N} \sum_{j=1}^{N} \left( P_{ij}^{t}-u_{t} \right)\left( P_{ij}^{o}-u_{o} \right)}{\sqrt{\left[ \sum_{i=1}^{N} \sum_{j=1}^{N} \left( P_{ij}^{t}-u_{t} \right)^{2} \right]\left[ \sum_{i=1}^{N} \sum_{j=1}^{N} \left( P_{ij}^{o}-u_{o} \right)^{2} \right]}},\#\left( S11 \right) \end{aligned}$$

where $P_{ij}^{o}$ and $P_{ij}^{t}$ correspond to the pixel values of phase ground truth and network output located at $\left( i,j \right)$ respectively, $N$ denotes the image size ($N$=256 in this case). The CC value ranges from 0 to 1 and 1 represents perfect correlation. These three metrics are usually used to assess image reconstruction quality compared to original image (ground truth) quantitively in different perspectives. We plot these three curves in Figure S3. In general, these values are reasonable and acceptable only that the variation is non-negligible. This problem can be attributed to afore-discussed local flaws. Therefore, in most cases, we prefer to adopt these three metrics to evaluate the performances of different trained models and we pay more attention to the mode accuracy than phase image metrics.


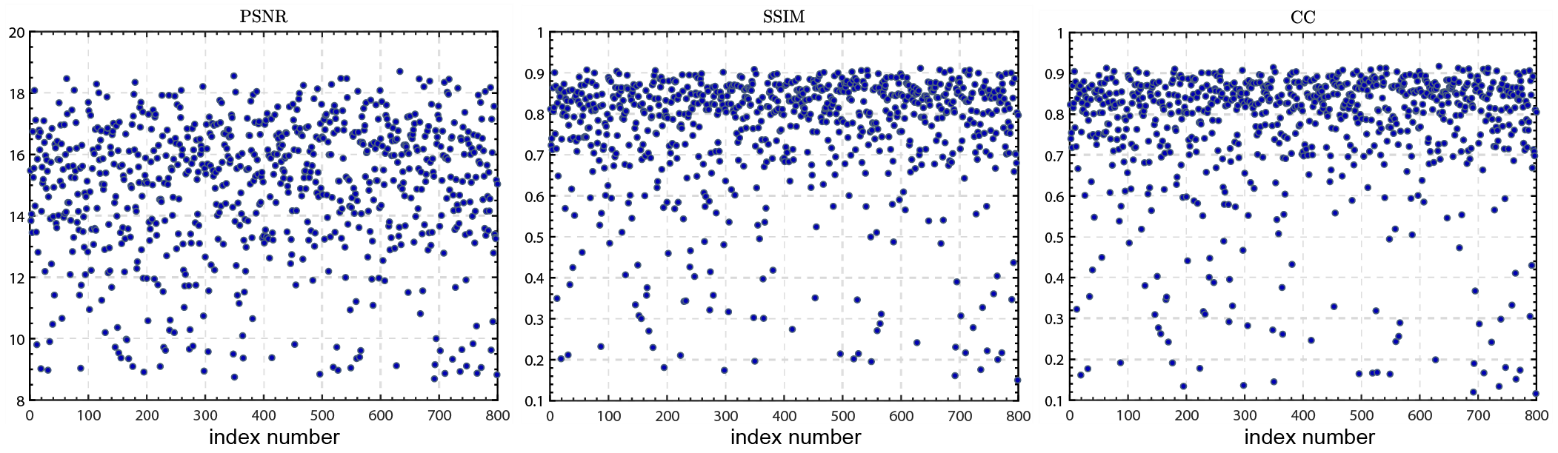


**Figure S3:** The PSNR, SSIM and CC metrics to evaluate testing results of VortexNet quantitively.

## Performance of VortexNet with only one intensity as input

We then illustrate how VortexNet performs when training the model with only the intensity at the focal plane. It is a strongly ill-posed problem and during the training process we find that the model is hard to converge indeed even with longer training time than that of other similar models. Though the training loss will decrease in fact, the validation loss does not go down as much. Of course the large amount of parameters inside the network enable itself to find some relationships between the intensity and the phase, after you feed massive data into the network, however, it is actually not learning but more like memorizing. This can be seen from the test results in Figure S4. We find that the modes accuracy declines radically. Many of the infer results tell the wrong $(Q,n_{0},M)$ , some of them even can not showcase their $(Q,n_{0},M)$ due to large-scale imperfections and the best modal we trained here is with an average value 11.79dB of PSNR, 0.53 of SSIM and 0.53 of CC. All in all, with just one more intensity as input, VortexNet can give really nice phase outputs.


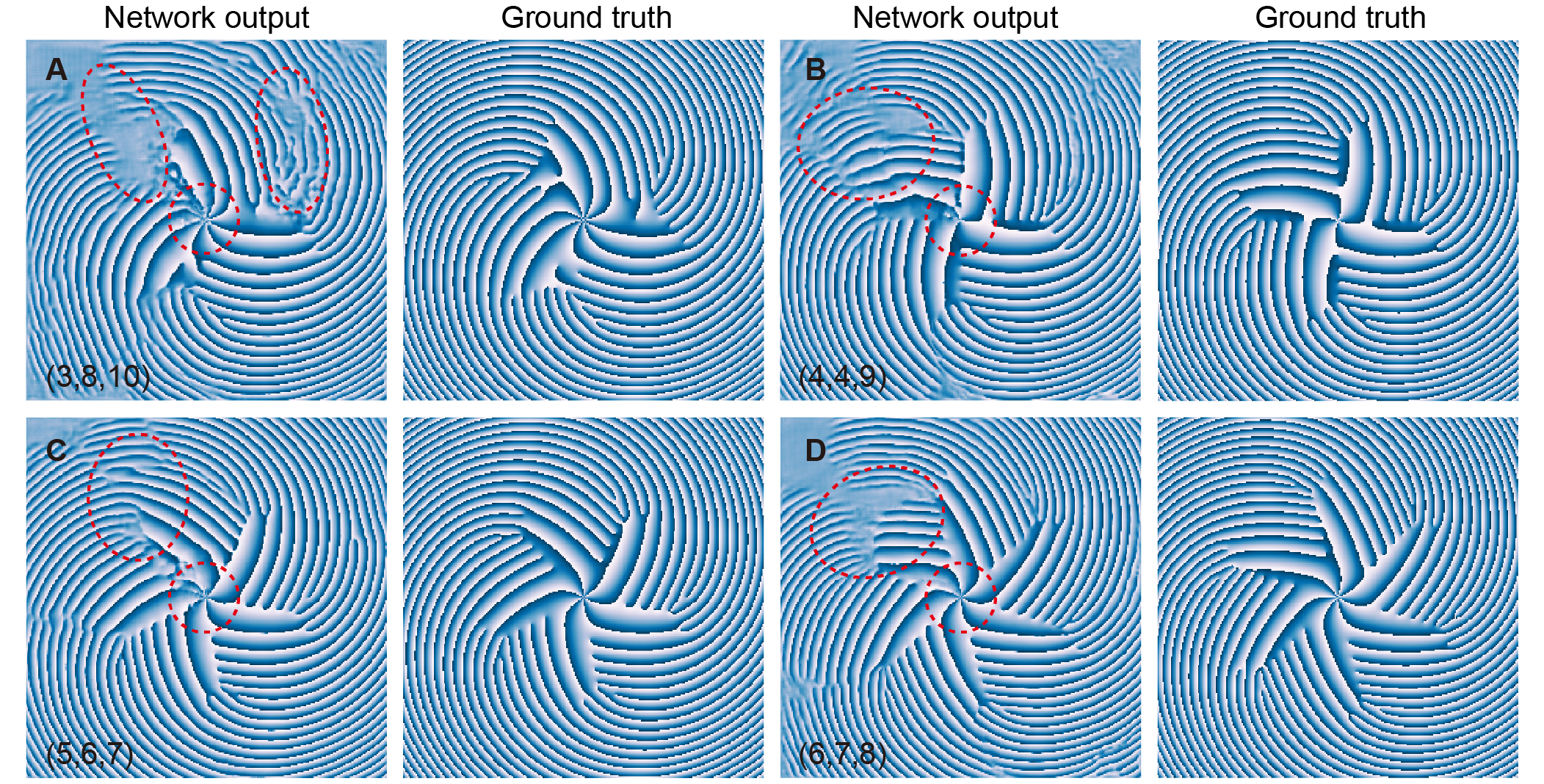


**Figure S4:** The performance of VortexNet when trained with only one intensity at the focal plane as input. Part of the artifacts are highlighted.

## LG modes & general phase objects results

To demonstrate the universal meaning of VortexNet, we then apply it to retrieve phase of familiar LG mode. Considering that the intensity distribution of LG mode is radially symmetric (single/multiple concentric rings) while the phase structure is rotationally symmetric, we find that the difference of data’s symmetry poses a great challenge for the network to converge based on several trials. Hence we break the radial symmetry to speed up the training phase by interfering the LG mode with a sphere wave, which can be easily implemented in experiments by adding two beam splitters. Meantime, the mature holography technique reminds us that the interference field contains the phase information sufficiently, therefore rather than taking two measurements as before, we in this case input the model with only one intensity pattern of interference field. To sum up, in above cases we measure twice (2 operations) while here we interfere first and measure once (also 2 operations), so the system and implementation are still concise enough. Indeed, the network converges rapidly and gives solid outputs as outlined in Figure S5(A)-(D). The digitally generated LG modes dataset contains 100 states with $p\in\left\{ 0,1,2,3,4 \right\}$ and $l\in\left\{ -10,-9,\ldots-1,1,\ldots,9,10 \right\}$ and each mode corresponds to 50 different initial phases where 43 of them are for training, 5 of them are for validation and 2 of them will be used during testing phase. The accuracy on test set reaches 100%. Besides, the trained model can recognize degenerate LG modes perfectly (See Figure S5 (A)-(B), Figure S5 (C)-(D)). It is reasonable because the resulting fields are different when $\psi_{p,l}^{LG}$ and $\psi_{p,-l}^{LG}$ interference with the same sphere wave respectively. This difference is also utilized in [11] recently.

Furthermore, we consider a more general case whereby the amplitude and phase are all “man-made”. We adopt randomly natural scene images as amplitudes and phases, in particular, the dataset comes from a computer vision benchmark dataset COCO [12]. We construct our dataset by selecting 10000 of them, transforming the RGB images to gray images, normalizing the amplitudes to 0~1 and phases to 0~$\pi$ respectively and cropping a 256×256 patch. We then fix this “light field” here as the first longitudinal position and propagate it via angular spectrum method to the second position. The defocused intensity at the second position is shown in the fourth column of Figure S5. We then feed two intensity images at different positions into the network and train it. The blind testing result can be seen in the fifth column of Figure S5, which indicates VortexNet can handle different situations.


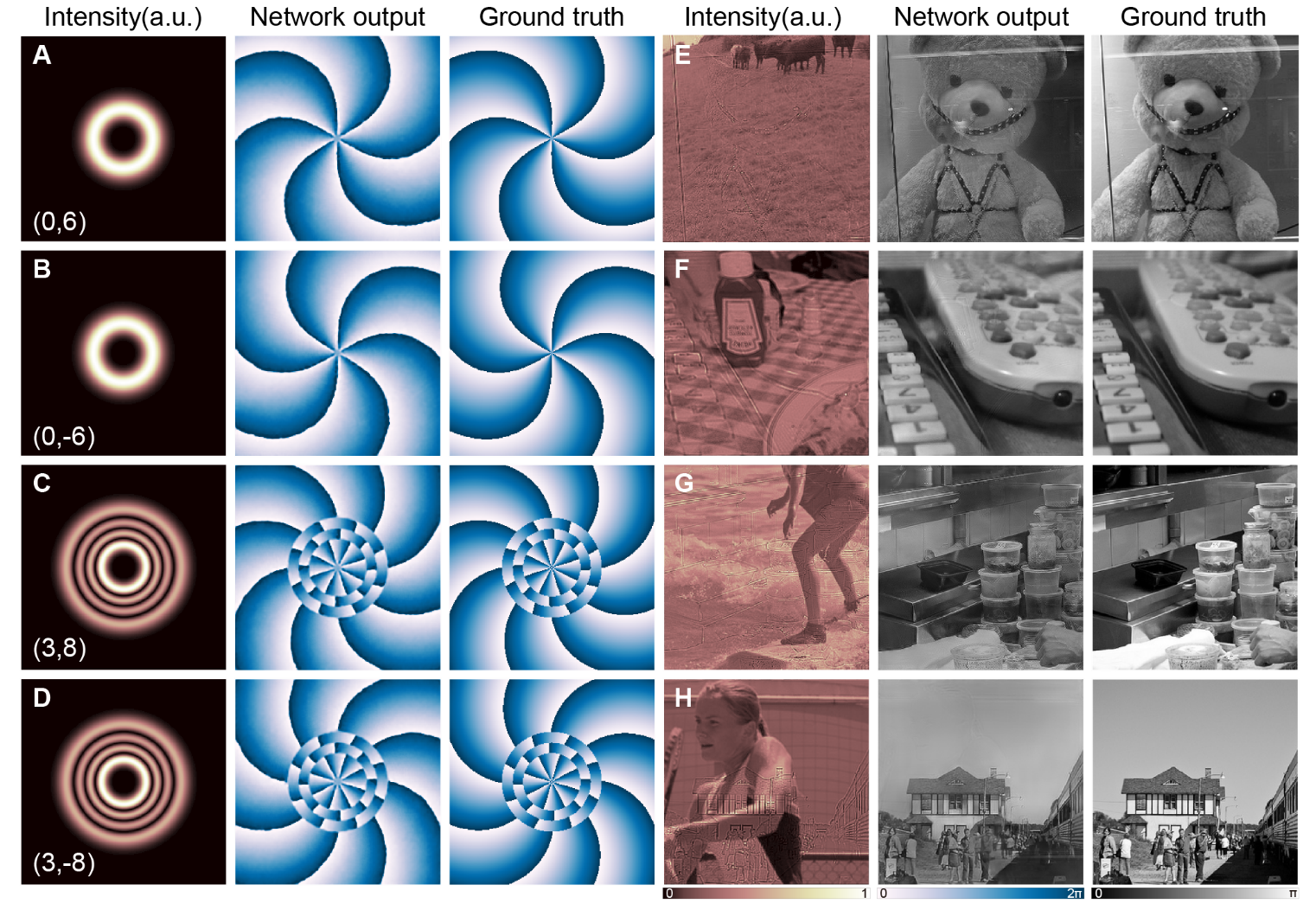


**Figure S5:** The performances of VortexNet on LG modes and general phase objects.

# The network structure, implementation and training details.

The VortexNet adapts the GAN framework [13] as depicted in Figure S6. In general, the generator consists of a downsampling path and an upsampling path with six and five blocks respectively. Each downsampling block is composed of two convolutional layers (kernel size 3×3, stride 1) with nonlinear layers (leaky rectified linear unit activation function or Leakey ReLU [14]) and a residual connection [15] with zero padding to compensate for the channel number mismatch, followed by a max pooling layer (2×2, stride 2, zero padding) to perform two-fold downsampling. The reason why the max pooling is chosen rather than average pooling is that we hope it can maintain or even magnify rather than lose the weak variance features between two adjacent modes. The upsampling block is similar but replacing the convolutional layers by transposed convolutional layers (kernel size 2×2, stride 2) when doing the upsampling. Besides it is without the residual connection and it concatenates the tensors of the last upsampling block with that of the corresponding symmetric downsampling block. The activation function of the last layer is modified to be tanh. The discriminator contains seven successive convolutional blocks. Each block of the first six has two convolutional layers (kernel size 3×3, stride 1) with Leaky ReLU activation layers and a downsampling convolutional layer (kernel size 3×3, stride 2). The last block has a convolutional layer (kernel size 4×4, stride 1) and a sigmoid activation layer. The input and output tensor size of the generator are 256×256×2, 256×256 respectively while for the discriminator, are 256×256 and 1. During the training phase, we hope the network can iteratively minimize the generator loss $l_{G}$ and the discriminator loss $l_{D}$, within which the $l_{G}$ is the weighted sum of pixelwise mean absolute error $l_{MAE}$ and the adversarial loss $l_{G,D,}$

$$\begin{aligned} l_{G}=\alpha l_{MAE}+\beta l_{G,D}.\#\left( S12 \right) \end{aligned}$$

In our case, $\alpha$is fixed to be 1.2 and $\beta$ is 1.0. The $l_{MAE}$ can be calculated as $l_{MAE}=mean\left[ G\left( x \right)-y \right]$, where $G(x)$ is the generator output when the input is $x$ and $y$ is the corresponding ground truth. The $l_{G,D}$ is determined by $l_{G,D}=\left[ D\left( G\left( x \right) \right)-1 \right]^{2}$ where $D\left( G\left( x \right) \right)$ denotes the discriminator evaluation when input is the “fake phase”. The $l_{G}$ is defined as

$$\begin{aligned} l_{D}=\frac{{D\left( G\left( x \right) \right)}^{2}+\left[ D\left( y \right)-1 \right]^{2}}{2},\#\left( S13 \right) \end{aligned}$$

where $D\left( y \right)$ is the discriminator output when input is label $y$. The training proceeds with learning rate $5\times{10}^{-5}$for generator, $5\times{10}^{-6}$ for discriminator using the Adam optimizer [16]. This is performed on a personal desktop with an Intel(R) Core™ i5-10400F central processing unit (CPU) @ 2.90 GHz and 16.0 GB random-access memory (RAM) as well as NVIDIA GeForce RTX 3060 Ti graphics processing unit (GPU). And the network is implemented using Pytorch 1.7.1 [17] with Python 3.8.5 and CUDA 11.1 environments. The batch size is 8 for training and 5 for validation and testing. As a result, the training phase normally lasts for about 25 hours till the validation loss drops down no more (See Figure S7 for recorded generator loss curves) and the inference time for one testing sample is about 0.02 seconds.


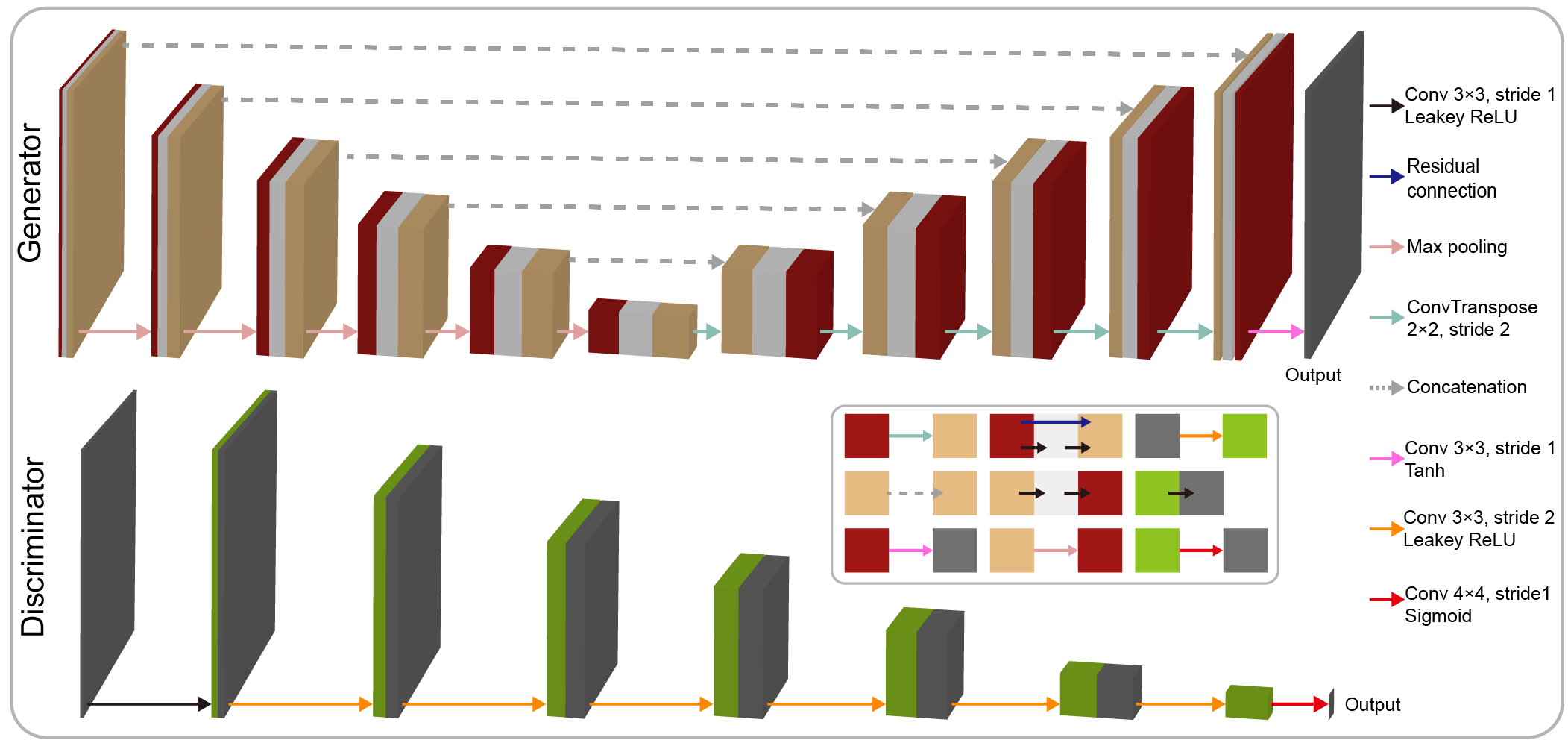


**Figure S6:** The VortexNet structure. It follows inherently a conditional GAN architecture and contains a generator and discriminator. Conv, convolutional layer; ConvTranspose, transposed convolutional layer.


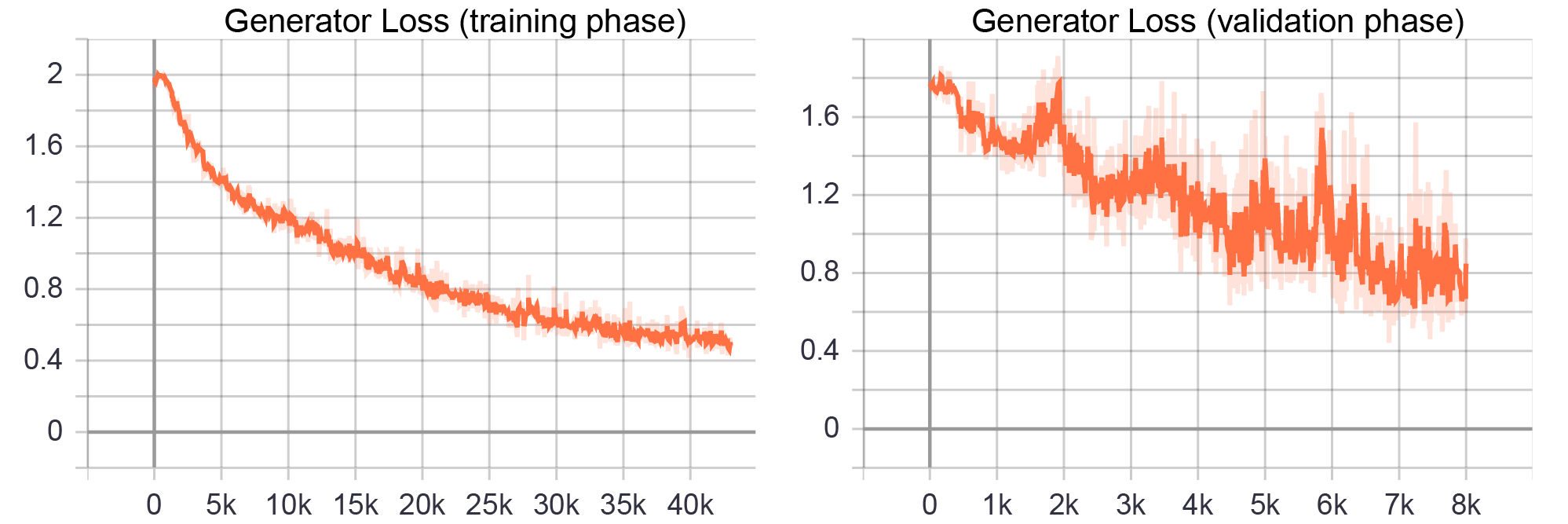


**Figure S7:** The loss curves of the generator during training and validation phase.

# The encoding/decoding rule of SU(2) modes and OSS analysis

The SU(2) vortex modes enjoy wealthy state space indeed so after defining specific encoding/decoding principles, they are highly suited to transmit information. Here we allocate the three parameters on an eight-bit binary number. More specifically, the parameter $Q$ takes values from four integers and corresponds to the top two bits, the parameter $n_{0}$ takes values from eight integers thus matches to the middle three bits and at last $M$ is similar to $n_{0}$ so it represents the last three bits. For example, SU(2) mode characterized by $\left( Q,n_{0},M \right)=(3,4,5)$ is equivalent to an eight-bit binary number $00011100$. See the following table for details.

**Table S1:** The corresponding relation between decimal mode parameters and binary numbers.

| Parameters | $Q$ | | | | $n_{0}$ | | | | | | | |
| --- | --- | --- | --- | --- | --- | --- | --- | --- | --- | --- | --- | --- |
| Decimal | 3 | 4 | 5 | 6 | 1 | 2 | 3 | 4 | 5 | 6 | 7 | 8 |
| Binary | 00 | 01 | 10 | 11 | 000 | 001 | 010 | 011 | 100 | 101 | 110 | 111 |
| Parameters | $M$ | | | | | | | |  |  |  |  |
| Decimal | 1 | 2 | 3 | 4 | 5 | 6 | 7 | 8 |  |  |  |  |
| Binary | 000 | 001 | 010 | 011 | 100 | 101 | 110 | 111 |  |  |  |  |

The optical secret key sharing scheme outperforms digitally counterpart in several aspects. First, we have to emphasize the optical signal is complex phasor and it characterizes *high parallelism* as well as *high speed*. It contains amplitude and phase simultaneously. Rather than distributing each shareholder a digital number (8-bit binary number in our case), we here send each shareholder a bundle of light. In this way, digital secret information is encoded into amplitude (which is difficult to interpret) while the secret can be decoded from the phase clearly. So the eavesdropper has to know the phase if he wants to stole the message. It means that he has to deal with the intensity-to-phase inverse problem, a well-known bugbear. The VortexNet offers an extra layer to protect the message. Besides, the sophisticated optical intensity/amplitude profiles complicate the secret information greatly and more fruitful multi-singularity modes e.g. higher dimensional SU(2) vortex beams are available to use to further enhance the capacity as well as complexity. Moreover, our protocol is scale-invariant benefitting from the intensity-based measurement, i.e. when the intensity measurement is multiplied by a constant number globally, it still can decrypt the message.

**References**

[1] Y. Shen, Y. Meng, X. Fu, and M. Gong, Wavelength-tunable Hermite&-Gaussian modes and an orbital-angular-momentum-tunable vortex beam in a dual-off-axis pumped Yb:CALGO laser, Opt. Lett. 43, 291 (2018).

[2] J. Pan, Y. Shen, Z. Wan, X. Fu, H. Zhang, and Q. Liu, Index-Tunable Structured-Light Beams from a Laser with an Intracavity Astigmatic Mode Converter, Phys. Rev. Applied 14, 044048 (2020).

[3] Y. Shen, X. Yang, X. Fu, and M. Gong, Periodic-trajectory-controlled, coherent-state-phase-switched, and wavelength-tunable SU(2) geometric modes in a frequency-degenerate resonator, Appl. Opt. 57, 9543 (2018).

[4] Y. Shen, X. Yang, D. Naidoo, X. Fu, and A. Forbes, Structured ray-wave vector vortex beams in multiple degrees of freedom from a laser, Optica 7, 820 (2020).

[5] Y. F. Chen, J. C. Tung, P. H. Tuan, and K. F. Huang, Symmetry Breaking Induced Geometric Surfaces with Topological Curves in Quantum and Classical Dynamics of the SU(2) Coupled Oscillators, Ann. Phys. 529, 1600253 (2017).

[6] Y. C. Lin, T. H. Lu, K. F. Huang, and Y. F. Chen, Model of commensurate harmonic oscillators with SU(2) coupling interactions: Analogous observation in laser transverse modes, Phys. Rev. E 85, 046217 (2012).

[7] Y. Shen, Z. Wang, X. Fu, D. Naidoo, and A. Forbes, SU(2) Poincare sphere: A generalized representation for multidimensional structured light, Phys. Rev. A 102, 031501 (2020).

[8] Y. F. Chen, C. H. Jiang, Y. P. Lan, and K. F. Huang, Wave representation of geometrical laser beam trajectories in a hemiconfocal cavity, Phys. Rev. A 69, 053807 (2004).

[9] L. Allen, M. W. Beijersbergen, R. J. C. Spreeuw, and J. P. Woerdman, Orbital angular momentum of light and the transformation of Laguerre-Gaussian laser modes, Phys. Rev. A 45, 8185 (1992).

[10] G. Wolberg, *Digital Image Warping* (IEEE Computer Society Press, 1994).

[11] L. Zhang, Y. Lin, Z. She *et al.*, Efficient sorting of orbital-angular-momentum states with large topological charges and their unknown superpositions via machine learning, arXiv:2105.13621 (2021).

[12] T.-Y. Lin, M. Maire, S. Belongie *et al.*, Microsoft COCO: Common Objects in Context. in *Computer Vision – ECCV 2014*, edited by D. Fleet *et al.* Springer International Publishing, Cham, (2014), pp. 740.

[13] Ian, J. Pouget-Abadie, M. Mirza *et al.*, Generative Adversarial Networks, arXiv:1406.2661 (2014).

[14] B. Xu, N. Wang, T. Chen, and M. Li, Empirical Evaluation of Rectified Activations in Convolutional Network, arXiv:1505.00853 (2015).

[15] K. He, X. Zhang, S. Ren, and J. Sun, Deep Residual Learning for Image Recognition. in *2016 IEEE Conference on Computer Vision and Pattern Recognition (CVPR)* (2016), pp. 770.

[16] Diederik and J. Ba, Adam: A Method for Stochastic Optimization, arXiv:1412.6980v3 (2017).

[17] A. Paszke, S. Gross, F. Massa *et al.*, PyTorch: An Imperative Style, High-Performance Deep Learning Library. in *NeurIPS* (2019).
